# 

**Published:** 1937

**Authors:** 


### CONTENTS.

| | Page |
|---|---|
| The Twenty-sixth Long Fox Memorial Lecture: The Debt of Western Medicine to the East. By V. B. Green-Armytage, M.D., F.R.C.P., F.C.O,G. | 239 |
| Child Guidance. By R. F. Barbour, M.B., Ch.B., M.R.C.P., D.P.M | 261 |
| A Lump in the Throat. By E. Watson-Williams, M.C., Ch.M., F.R.C.S. | 279 |
| The Bristol Royal Infirmary Bicentenary | 287 |
| Reviews of Books | 301 |
| Editorial Notes | 313 |
| Meeting of Societies | 318 |
| The Medical Library of the University of Bristol | 321 |
| Publications Received | 323 |
| Local Medical Notes | 323 |

